# Changes in Toe Clearance Due to Adjusting the Dorsiflexion Angle of Ankle–Foot Orthoses: A Study in Healthy Individuals

**DOI:** 10.3390/bioengineering11121293

**Published:** 2024-12-20

**Authors:** Hiroshi Hosokawa, Fumiaki Tamiya, Ren Fujii, Ryu Ishimoto, Masahiko Mukaino, Yohei Otaka

**Affiliations:** 1 Department of Rehabilitation Medicine, Musashigaoka Hospital (Tanakakai Medical Corp.), Kumamoto 861-8003, Japan; 2 Musashigaoka Clinical Research Center, Musashigaoka Hospital (Tanakakai Medical Corp.), Kumamoto 861-8003, Japan; 3 Department of Rehabilitation Medicine, School of Medicine, Fujita Health University, Toyoake 470-1192, Japan; 4 Department of Rehabilitation, Musashigaoka Hospital (Tanakakai Medical Corp.), Kumamoto 861-8003, Japan; 5 Graduate School of Comprehensive Human Sciences, University of Tsukuba, Tsukuba 305-8575, Japan; 6 Department of Rehabilitation Medicine, Ibaraki Prefectural University of Health Sciences Hospital, Ami 300-0394, Japan; 7 Department of Rehabilitation Medicine, Hokkaido University Hospital, Sapporo 060-8648, Japan

**Keywords:** ankle–foot orthoses, toe clearance, gait analysis

## Abstract

This study investigated the effects of ankle dorsiflexion angle adjustments in ankle–foot orthoses (AFOs) on the gait of healthy individuals. Fifteen healthy participants engaged in treadmill walking tasks while wearing AFOs with dorsiflexion angles set at 0°, 5°, 10°, and 15°. Three-dimensional treadmill gait analysis was used to collect data during treadmill walking. The analysis focused on toe clearance and the contribution of the vertical component of limb shortening (LS), and compared them across different dorsiflexion angles. The results indicated a significant increase in toe clearance at 10° (median [interquartile change]: 5.03 [0.90] vs. 5.98 [1.18], *p* < 0.01) and 15° (5.03 [0.90] vs. 5.82 [1.11], *p* < 0.01) dorsiflexion angle conditions compared to the 0° condition. Similarly, LS demonstrated significant increases at 10° (4.89 [1.97] vs. 5.87 [1.31], *p* < 0.01) and 15° (4.89 [1.97] vs. 5.61 [1.65], *p* < 0.01) conditions when compared with the 0° condition. These findings support the notion that higher dorsiflexion angles in AFOs lead to increased toe clearance and LS, suggesting the effectiveness of dorsiflexion angle adjustments as a strategy to address reduced toe clearance.

## 1. Introduction

Maintaining adequate toe clearance during the swing phase of a gait is crucial in assessing the risk of falls, as it relates directly to tripping incidents, a primary factor contributing to falls [1]. In healthy individuals, toe clearance is primarily achieved through limb shortening (LS), which depends on hip and knee flexion as well as ankle dorsiflexion [2]. Previous studies have shown that individuals with motor impairments often experience disturbances in these movements, negatively affecting their ability to achieve toe clearance [3,4,5,6,7,8]. Consequently, toe clearance has become an important focus of rehabilitation to reduce fall risk in patients with motor impairments [9,10]. A common response among these patients to reduced toe clearance is the use of compensatory movements, such as hip elevation and circumduction [2,11,12]. While these strategies can enhance toe clearance, their associated increase in energy expenditure [5] highlights the importance of minimizing excessive reliance on them during rehabilitation.

In clinical situations, ankle–foot orthoses (AFOs) are frequently prescribed by medical doctors for patients with paresis caused by conditions such as stroke or peroneal nerve injury to improve gait performance by enhancing stability, elevating toe clearance, and reducing compensatory movements [13,14,15]. AFOs have been shown to impact LS during swinging, leading to enhanced toe clearance and reduced compensatory movements, such as hip elevation [16]. These effects of the AFO on toe clearance may also be related to the angle of the AFO, considering the numerous studies on ankle angles during swing and toe clearance [2,17]. While adjusting the dorsiflexion angle of the AFO is a common practice, the specific conditions and extent of this effect remain underexplored in clinical settings. Consequently, further research is necessary to identify optimal orthotic adjustments that maximize the effects of AFOs on gait patterns.

To investigate the impact of AFO settings on toe clearance, this study examined the relationship between toe clearance and the dorsiflexion angle of AFOs. Using three-dimensional gait analysis, we evaluated the gait performance of healthy participants wearing AFOs set at various angles, focusing on toe clearance and joint kinematics during the swing phase. The hypothesis was that increasing the dorsiflexion angle of the AFO would enhance toe clearance, accompanied by greater LS.

## 2. Materials and Methods

### 2.1. Participants

Fifteen healthy male subjects were recruited for this study. Participants were recruited through hospital bulletin boards. Inclusion criteria were (1) age ≥ 18 years and (2) provision of written informed consent. The exclusion criterion was a history of neurological or orthopedic conditions. Screenings were conducted through non-structured interviews and physical examinations, and all recruited individuals met the eligibility criteria. All participants provided written informed consent before participation. This study was approved by the Medical Ethics Committee of Fujita Health University.

### 2.2. Experimental Setup and Conditions

Gait measurements were performed using a three-dimensional treadmill gait analysis system (KinemaTracer^®^; Kissei Comtec Co., Ltd., Matsumoto, Japan). This system included a PC for data recording and analysis, along with four charge-coupled device cameras with 60 Hz frame rates, strategically installed around both sides of the treadmill. The cameras recorded three-dimensional coordinates (X: left/right, Y: front/back, Z: up/down) of markers placed on various body landmarks. These markers included the bilateral acromions, hip joints (1/3 of the greater trochanter on the line connecting the superior anterior iliac spine and greater trochanter), knee joints (midpoint of the anteroposterior diameter of the lateral femoral epicondyle), ankle joints (external capsule), toes (fifth metatarsal head), and iliac crests (located on a vertical line through the hip joint), totaling 12 markers.

The AFOs used in this study were adjustable posterior strut AFOs (APS-AFOs). An APS-AFO is an articulated AFO used for gait rehabilitation in patients [18,19,20]. The APS-AFO facilitates easy adjustment of the ankle-hinge joint, enabling customization of the strut’s length and thickness to accommodate the patients’ needs. In this study, the hardest aluminum strut was used. The AFO was affixed to the right lower leg. The difference in leg length between the left and right legs was measured with a caliper and adjusted using insoles. The dorsiflexion angle of the AFO was adjusted to four fixed positions: 0°, 5°, 10°, and 15°. The order of measurements for the different angle settings was randomized to minimize bias. A rest period of 5–10 min was provided before each measurement. During treadmill walking tasks, the treadmill speed was set to a comfortable pace, which was determined before the session. The starting pace was at a minimum of 70% of the participant’s self-selected comfortable walking speed, which was measured over a 10 m distance on level ground beforehand. Data collection was conducted for a duration of 20 s.

### 2.3. Data Analysis

This study analyzed the following gait analysis parameters: gait cycle time, stance and swing time of the right limb, double stance time, stride and step length, joint angles (hip, knee, and foot) during the swing phase, and toe clearance with its components. Toe clearance and its components were calculated according to toe clearance analysis methodology reported previously [16,21,22,23]. Briefly, toe clearance was calculated as the vertical raise of the toe at midswing compared to midstance. The components of swing-related movements contributing to toe clearance were also calculated. These included the vertical component of the LS, further subdivided into knee joint and ankle joint contributions, as well as the vertical components of compensatory movements, such as pelvic obliquity, stance-side hip elevation, and foot elevation due to circumduction. The timings of the midswing and midstance were defined as those moments when the toe marker was aligned directly beneath the hip marker during the swing and stance phases, respectively.

The normality of the data distribution was evaluated using the Shapiro–Wilk test. For differences between the ankle angle settings of the AFO, repeated measures ANOVA was applied to normally distributed data, while the Friedman test was used for non-normally distributed data. Post hoc comparisons were conducted using paired *t*-tests for normally distributed data and the Wilcoxon signed-rank test for non-normally distributed data. The Bonferroni method was employed to adjust for multiple comparisons. Statistical analyses were performed using R software (version 4.2.1), with the significance level set at 5%.

## 3. Results

The ages of the 15 participants were 32.4 (SD: 5.4) years, with an average height of 171.9 (4.9) cm and weight of 65.3 (5.5) kg. The median gait speed on the treadmill was 3.80 (1.25) km/h.

Spatiotemporal parameters are listed in Table 1. No significant differences were observed among conditions ranging from 0° to 15°. The maximum angles of each joint during the swing phase are listed in Table 2. Only ankle dorsiflexion and plantar flexion differed significantly between conditions.

**Figure 1:**
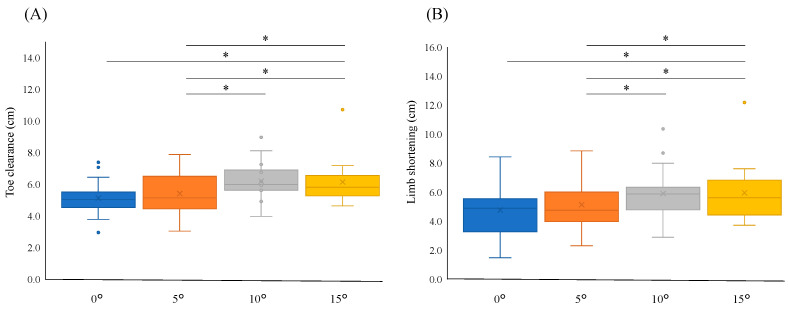
Box plots of toe clearance and the vertical component of limb shortening (LS) in relation to the ankle angle. (**A**) Toe clearance at ankle angles 0°, 5°, 10°, and 15°; and (**B**) vertical displacement by LS at ankle angles 0°, 5°, 10°, and 15°. The edges of the box plot correspond to the 25th and 75th percentiles. * *p* < 0.05.

The Friedman test was employed to assess differences in toe clearance across various dorsiflexion angles of the AFO, revealing significant findings (*p* < 0.001). Detailed comparisons of the AFO (right) side indicated significant differences between 0° and 10° (median [interquartile change]: 5.03 [0.90] vs. 5.98 [1.18], *p* < 0.001), 0° and 15° (5.03 [0.90] vs. 5.82 [1.11], *p* < 0.001), 5° and 10° (5.14 [1.57] vs. 5.98 [1.18], *p* = 0.002), and 5° and 15° (5.14 [1.57] vs. 5.82 [1.11], *p* = 0.012) (Figure 1A).

Similarly, the total LS, based on the dorsiflexion angles of the AFO, exhibited significant variations (*p* < 0.001). Significant differences were observed in comparisons between 0° and 10° (4.89 [1.97] vs. 5.87 [1.31], *p* < 0.001), 0° and 15° (4.89 [1.97] vs. 5.61 [1.65], *p* < 0.001), 5° and 10° (4.75 [1.71] vs. 5.87 [1.31], *p* = 0.026), and 5° and 15° (4.75 [1.71] vs. 5.61 [1.65], *p* = 0.016), as detailed in Figure 1B.

**Figure 2:**
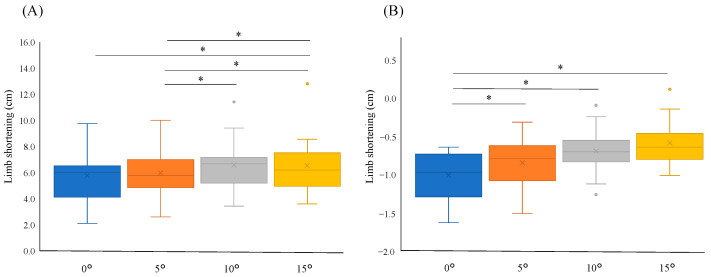
Box plot illustrating the vertical component of limb shortening (LS) due to knee and ankle movement in relation to the ankle angle. (**A**) Vertical displacement by knee component at ankle angles 0°, 5°, 10°, and 15°; and (**B**) vertical displacement by ankle component of LS at ankle angles 0°, 5°, 10°, and 15°. The edges of the box plot correspond to the 25th and 75th percentiles. * *p* < 0.05.

The limb-shortening component contributing to toe clearance can be subdivided into knee and ankle components. Dorsiflexion angle adjustments significantly affected both the knee and ankle components of LS (*p* = 0.001 and *p* < 0.001, respectively). Specific angles, such as 0° to 10° (6.01 [2.24] vs. 6.68 [1.82], *p* = 0.008), 0° to 15° (6.01 [2.24] vs. 6.20 [1.82], *p* = 0.016), 5° to 10° (5.79 [1.89] vs. 6.68 [1.82], *p* = 0.006), and 5° to 15° (5.79 [1.89] vs. 6.20 [1.82], *p* = 0.026), demonstrated significant differences in the knee component of LS, whereas the ankle component showed significant differences between 0° and 5° (−0.97 [0.48] vs. −0.79 [0.37], *p* = 0.013), 0° and 10° (−0.97 [0.48] vs. −0.70 [0.26], *p* = 0.007), and 0° and 15° (−0.97 [0.48] vs. −0.64 [0.30], *p* = 0.004), as shown in Figure 2.

## 4. Discussion

This study aimed to investigate the impact of ankle dorsiflexion angle settings of AFOs on gait. After comparing the settings, significant differences were observed primarily in toe clearance and the contribution of LS (total, knee, and ankle) to toe clearance during the swing phase of the gait. In particular, significant increases in toe clearance and LS were observed at dorsiflexion angles 10° and 15° compared with the baseline 0° condition. Notably, this significant difference was evident in the knee and ankle components of LS.

The primary focus of this study was to explore the relationship between ankle dorsiflexion angle facilitated by AFOs and toe clearance in healthy individuals. During the swing phase, toe clearance is primarily achieved through LS, which is influenced by ankle dorsiflexion, as well as hip and knee flexion. Our findings indicated that increasing the dorsiflexion angle enhanced toe clearance by promoting greater LS. These results are consistent with those of previous studies demonstrating the direct impact of ankle dorsiflexion on improving toe clearance [2].

In addition to the direct effect of dorsiflexion, an AFO can also indirectly impact knee flexion [24]. In this study, the increased dorsiflexion by the AFO led to an increase in the knee component of LS, possibly enhancing this effect. Although the present study did not show a significant effect of the AFO’s maximum angle itself, there was an increase in the vertical displacement due to the knee-derived component of LS. This may be attributed to the timing of knee flexion, which is influenced by the ankle angle. The indirect knee flexion arising from forced ankle dorsiflexion at ground contact by the AFO may shorten the timing of knee flexion, affecting knee-derived limb shortening at midstance. On the other hand, excessive forced knee flexion during the stance phase may compromise stability in patients with severe paresis. Although the current results indicate no negative effects on gait stability parameters due to the ankle angles, the impact on gait performance should be evaluated from multiple perspectives within the patient population.

Despite the overall positive impacts of an AFO on gait performance in paretic patients, it can have negative effects if it does not properly match the individual’s motor function. In a previous study investigating the effects of AFOs in healthy individuals, significant reductions were reported in gait speed, cadence, number of steps, and stride length, as well as decreased forward propulsion and dynamic stability, in the AFO condition compared to the no-AFO condition [25]. Even in patients with mild paresis, a negative impact on gait performance due to AFOs can be observed, although it appears to be negligible in those with severe paresis [23]. These effects may be attributed to restricted ankle motion and diminished sensory feedback in the foot, which are believed to adversely affect the time–distance factor and kinematic factors in the stance phase, thereby increasing gait instability. Our study did not observe significant changes in these factors across 0–15° dorsiflexion conditions, suggesting that the adverse effects of AFOs resulting from restricted ankle motion may not be impacted by the angular settings. However, this should be verified under conditions with varying levels of paresis.

### Limitations

This study had a few limitations. First, this study was conducted exclusively in healthy individuals. The negative impacts of the AFO angle, such as instability due to excessive knee flexion, which is related to reduced motor ability, may not have been fully captured by this study. Further research involving individuals with hemiparesis is necessary to better understand the impact of the AFO angle on gait performance. Second, the sample size was relatively small. While the analysis identified significant trends, the sample size may have been too small to detect differences between minor angle adjustments. A larger sample size encompassing a larger patient population would provide a more robust understanding of the observed effects. Third, the present study was conducted on the treadmill, which may have influenced the results. The generalizability of the results to overground walking should be further confirmed. However, considering the similarity in kinematic patterns between overground walking and treadmill walking [26], its impact on the kinematic difference in toe clearance strategy between different ankle angle conditions should be minimal. Lastly, the present study used APS-AFOs, which are less common than conventional or thermoplastic AFOs. However, the similarity in effect on toe clearance strategy between thermoplastic AFOs and APS-AFOs supports the generalizability of the results [16].

## 5. Conclusions

This study highlights a close relationship between the dorsiflexion angle of AFOs and toe clearance, showing that increased dorsiflexion enhances toe clearance through the contribution of the ankle angle-related LS component. These findings support the clinical utility of adjusting the dorsiflexion angle of AFOs as a strategy to improve toe clearance. By visualizing trends in dorsiflexion angle adjustments and their effects on toe clearance components, this study lays a foundation for targeted gait improvement interventions using AFOs. Further research involving patients with gait impairments would enhance our understanding of the practical utility of adjusting AFO settings in real clinical practice.

## Figures and Tables

**Table 1:** Spatiotemporal parameters at each ankle angle condition [median (interquartile range)].

	0°	5°	10°	15°	*p*
Gait cycle time (s)	1.13 (0.19)	1.11 (0.19)	1.08 (0.15)	1.09 (0.20)	0.561
CV of Gait cycle time (s)	1.79 (0.59)	2.22 (0.68)	2.11 (0.84)	1.79 (0.43)	0.212
Stance time (s)	0.76 (0.13)	0.73 (0.15)	0.72 (0.10)	0.71 (0.11)	0.931
Swing time (s; AFO side)	0.38 (0.03)	0.38 (0.05)	0.36 (0.05)	0.37 (0.06)	0.781
Single stance time (s; AFO side)	0.37 (0.04)	0.36 (0.05)	0.35 (0.05)	0.35 (0.05)	0.976
Double stance (s)	0.19 (0.06)	0.18 (0.07)	0.16 (0.06)	0.16 (0.05)	0.153
Stride length (s)	115.85 (24.88)	121.70 (27.91)	121.56 (28.56)	117.92 (30.84)	0.523
CV of stride length (%)	2.19 (1.14)	2.35 (0.42)	2.61 (1.01)	2.57 (0.9)	0.622
Step length (cm)	57.80 (12.49)	59.52 (15.06)	58.43 (15.08)	56.64 (17.10)	0.791

CV, coefficient of variation; AFO, ankle–foot orthosis.

**Table 2:** Maximum joint angles during swing phase at each condition [median (interquartile range)].

	0°	5°	10°	15°	*p*
AFO side (°)					
Hip flexion	29.31 (6.87)	32.28 (7.63)	32.50 (6.79)	32.44 (4.82)	0.831
Hip extension	1.76 (8.96)	1.10 (6.12)	−1.54 (7.42)	−0.95 (6.71)	0.793
Knee flexion	59.14 (4.30)	59.85 (6.65)	58.82 (8.46)	58.21 (6.29)	0.582
Knee extension	4.90 (9.15)	7.24 (10.74)	7.41 (10.85)	6.70 (8.61)	0.601
Ankle dorsiflexion	−4.06 (5.06)	−0.58 (2.88)	−0.52 (3.80)	−1.22 (2.44)	<0.001
Ankle plantar flexion	9.13 (2.68)	7.43 (5.06)	7.18 (4.73)	7.58 (3.56)	<0.001
Usual side (°)					
Hip flexion	27.55 (7.11)	29.11 (4.95)	29.68 (4.82)	31.74 (4.67)	0.127
Hip extension	6.07 (10.64)	4.43 (8.14)	5.64 (8.44)	5.23 (6.83)	0.992
Knee flexion	62.78 (8.29)	62.61 (7.39)	61.76 (10.47)	61.80 (4.84)	0.791
Knee extension	5.88 (14.99)	6.14 (12.42)	6.36 (13.86)	10.21 (16.60)	0.582
Ankle dorsiflexion	−0.40 (8.69)	−0.18 (7.92)	0.14 (7.32)	1.81 (8.23)	0.683
Ankle plantar flexion	21.90 (7.28)	21.47 (6.58)	18.75 (5.86)	19.51 (11.14)	0.471

AFO, ankle–foot orthosis.

## Data Availability

Datasets used and/or analyzed during the current study are available from the corresponding author upon reasonable request.
